# Elucidating the Paradox of Regulating Environmental Sustainability (Mis)management and Motivations: The Case of Thai Fisheries

**DOI:** 10.1007/s00267-022-01689-6

**Published:** 2022-07-23

**Authors:** Vinh Sum Chau, Montita Bunsiri

**Affiliations:** Kent Business School, University of Kent, Canterbury, Kent, CT2 7FS UK

**Keywords:** Configuration theory, Sustainability management, Fishing regulations, Environmental management, Thai community

## Abstract

This article introduces and elucidates a new sustainability management paradox by examining the difficulties of applying the European Union’s illegal, unreported and unregulated (IUU) fishing regulations in Thai waters. Interviews were conducted with key stakeholders of Thailand’s fishery sector to explore the particularities of the area. Configuration theory—from a strategic management perspective—was used to guide empirical research and extend it to the context of environmental regulation. The research finds that when it makes more business sense for stakeholders to engage in sustainability matters, more explicit engagement might take place of the wrong type, but the true sustainability objectives become performed more poorly and mismanaged, perhaps resulting in a worse-off position than started with. This is because regulation is astute at setting targets, but ineffective at engaging with key stakeholders. A composite model of how configuration theory fits within discussions of sustainability motivations is posited as the theoretical contribution to knowledge.

## Introduction

A predicament has been identified in recent research into the management and motivations of environmental sustainability: the misalignment of the intentions of regulatory policy makers and the stakeholders expected to carry them out (e.g. Schaltegger and Burritt 2018). This is because these policy objectives may not be a key part of the stakeholder’s mission or a firm is too small to partake in any efforts to support environmental sustainability, notwithstanding any evidence for any causal link to their accomplishment (Weber 2008). While environmental regulation may influence a firm to invest in abatement technologies relating to that sustainability issue (Rodriquez Lopez et al. 2017), for global concerns, the motivation to participate in such objectives requires international collaborative efforts (Singh and Lakhan 1989). Thus, a renowned view that effective environmental regulation will lead to improved innovation—known as the Porter Hypothesis—is much challenged (e.g. Lanoie et al. 2011). While much of these successes concern the make-up of key actors’ business models that incorporate these regulations (Schaltegger et al. 2016), there often remains a black box situation of not knowing how regulatory policies work in unknown and closed settings (e.g. Chau and Witcher 2005). In a similar vein, sustainability values also need to be ingrained into an entirety of stakeholders concerned if they are to be taken seriously and stand a chance of being effective (Haffar and Searcy 2019).

A prominent knowledge base for understanding how various stakeholders respond to a situation, such as an environmental sustainability concern, is configuration theory—that is to say, the configuration into which the stakeholders fit determines a likely action. This is because of various constraints an actor might be facing—for example, a family-orientated firm possesses a lower level of social performance due to fewer formal ethical codes to abide by (Cuadrado-Ballesteros et al. 2017). While configuration theory has demonstrated positive correlation with a firm’s performance (Pinto and Curto 2007), and organizational typing might shed light on environmental innovations (Russo-Spena et al. 2021), studies have yet to extend configurations beyond conventional “firms” to a broader group of stakeholders (for example, a public officer) within a sector to understand a broader extent of the constraints of managing environmental sustainability issues. Maniora (2018) talked specifically about two archetypes (prospectors and defenders) of the Miles and Snow (1978) configuration and their likely approach to sustainability (mis)management. The present research extends this argument to relate all four archetypes of the Miles and Snow framework to more stakeholders of a sector, thereby penetrating other key actors within the broader market, in support of the view that pursuing market-based approaches to environmental management is favorable (Konefal 2013). As the theory argues that stakeholders (companies, individuals, and/or otherwise) respond strategically to concerns about how best to organize and manage work due to individual motivations, our categorization of the stakeholders of the Thai fishery sector into the archetypes will offer useful understanding of the tendency and/or success for/of implementing new regulatory policies. Specifically, our grouping of the key stakeholders for the context of the regulatory control will enable us to determine how risks are taken along with the decisions and issues faced, to determine a type of strategy to bring about success. Thus, the present research seeks to answer: what is the impact of the imposition of externally imposed sustainability objectives on a sector of environmental concern, and how can configuration theory be used to understand the response of its key stakeholders? Filling in this knowledge gap will help future regulators enact more appropriate regulations across cultures and settings and allow local implementors direct efforts to stakeholders with better effect. This research is only one new pathway toward achieving environmental sustainability, but its approach is novel, and may be applied to numerous sectors that share similar conditions of regulatory imposition and contexts of environmental concern.

Acknowledging the complex balancing of cost and sustainability management in declining global fish stocks (Pontecorvo and Schrank 2014; Longo and Clausen 2011), we elucidate and offer insights on this paradox in which increasing efforts to manage regulatory environmental and sustainability objectives may not improve their achievement. The context of the present study is the unique fishery sector in Thailand, as it represents a situation in which the then newly enacted local fishing legislation to incorporate important international regulatory standards had not taken into consideration the constraints suffered by all the stakeholders in that fishing region of Thailand as part of the planning process. The specific international regulatory standard of concern is the European Council Regulation No 1005/2008, enacted by the European Union, which extends to Thai waters, but was implemented with minimal consultation with international partners or understanding of specific regional constraints.

The present article is structured as follows: it first provides the context of the Thai fishery sector and relates it to current thinking in configuration theory, and outlines how sustainability management models have related to this area. It then explains the methodology for the primary research including details of data collection and analysis of responses from key stakeholders in the Andaman Coast of Thailand. It then presents key constraints identified by the key stakeholders as empirical findings, before discussing these within a composite framework of configurations. It finally concludes with opinions on the effectiveness of stakeholder management within the Thai fishery sector, and suggests how the findings and ideas have generalizable value in similar contexts that may benefit from using configuration theories to group broad ranging stakeholders.

## Sustainability Management and Configuration Theory for Thailand Fisheries

### Thai Fishery Sector

Thailand is the 10th highest producing country in 2019 with exports valued around USD 5.8 billion, positioning it sixth in the world of major fish exporting countries (FAO 2021). Most of the catch is usually exported to the USA, Japan, Australia, Canada and the UK (Agriculture and Agri-Food Canada 2015). Approximately 60% of total marine catch in Thailand occurs in Thai waters and the rest from outside the Thai Exclusive Economic Zone (FAO 2021). The two types of fisheries in Thailand are small-scale fisheries and large, industrial-scale fisheries. The former use non-powered, outboard-powered and inboard-powered vessels of less than ten gross tonnages (Teh et al. 2015) performed to support local families and daily lives (i.e. for subsistence), but can also be for commercial purposes. The latter are capital-intensive fisheries, using relatively large vessels exceeding ten gross tonnages with a high degree of mechanization, amounting to ~90% of marine catch, specifically for commercial purposes (FAO 2022). Both are important, as in rural areas, fish contributes an affordable source of animal protein and maintains food security, but there is often an ignorance of the importance of small-scale fisheries, and the figures are possibly underestimated to around two times (Teh and Pauly 2018).

Fisheries are a common resource pool but their availability for the next users will be diminished when they are exploited by the previous generation of users, and the exact extent of impact is difficult to determine due to measurement and monitoring constraints (Schijns and Pauly 2021). The fish supply serving the market is not easily controlled or managed due to the complex fish and marine migratory behavior, together with hard to measure and inaccurate stock assessments (Freire et al. 2020). Moreover, the variety of fishing vessels and gear used have affected ecosystems in different ways, often not recognizing the impact of smaller fishers (Palomares and Pauly 2019). So, not only is the nature of the industry itself complicated, the complexities of fishing are also affected by biological, social, economic and the environmental sectors playing their different parts (Dudley 2006). For the past three decades, fishery resources and environment sustainability have been threatened by “overfishing” (Zeller and Pauly, 2019)—an amount that breaches “a level of catch that ensures the long-term existence of the fish species and prevents their depletion” (Department of European Affairs 2016a, p. 2). This applies to small-scale fisheries as well, albeit regulation and guidance have often been on a voluntary best-practice basis (e.g. FAO 2015, 2022).

For this reason, the problem of illegal (activities that infringe regulations or are absent of a country’s permission), unreported (activities that are not reported or misreported) and unregulated (activities that violate fisheries conservation and management measures) fishing is now a key concern of the Thai fishery sector. This is referred simply as IUU fishing from hereon (for definitions and further technical details, see Panjarat 2008; Schmidt 2004; Doulman 2004). IUU fishing is seen as a key barrier to fisheries sustainability (Coppa et al. 2021), but placing blanket protections on specific areas of fishing decline may not be effective due to localized constraints (Relano et al. 2021). Specifically, Thailand had been controlled by numerous legislative and regulatory mechanisms in recent years (see Suebpala et al. 2015; FAO 2001, 2002), in particularly the European Union’s Council Regulation (EC) No 1005/2008, under the Common Fisheries Policy known as the EU IUU Fishing Regulations (Regulation 2013). These regulations control the movement of fishery products entering the EU market as well as enhance global sustainability of fisheries (Lutchman et al. 2001). A “yellow card” warning was issued to Thailand on 21 April 2015 (European Commission 2015a) for ineffective national control of IUU fishing (European Commission 2015b; Gotev 2015) within the regulation, although this has been lifted in January 2019 (see Kadfak and Antonova 2021).

**Fig. 1:**
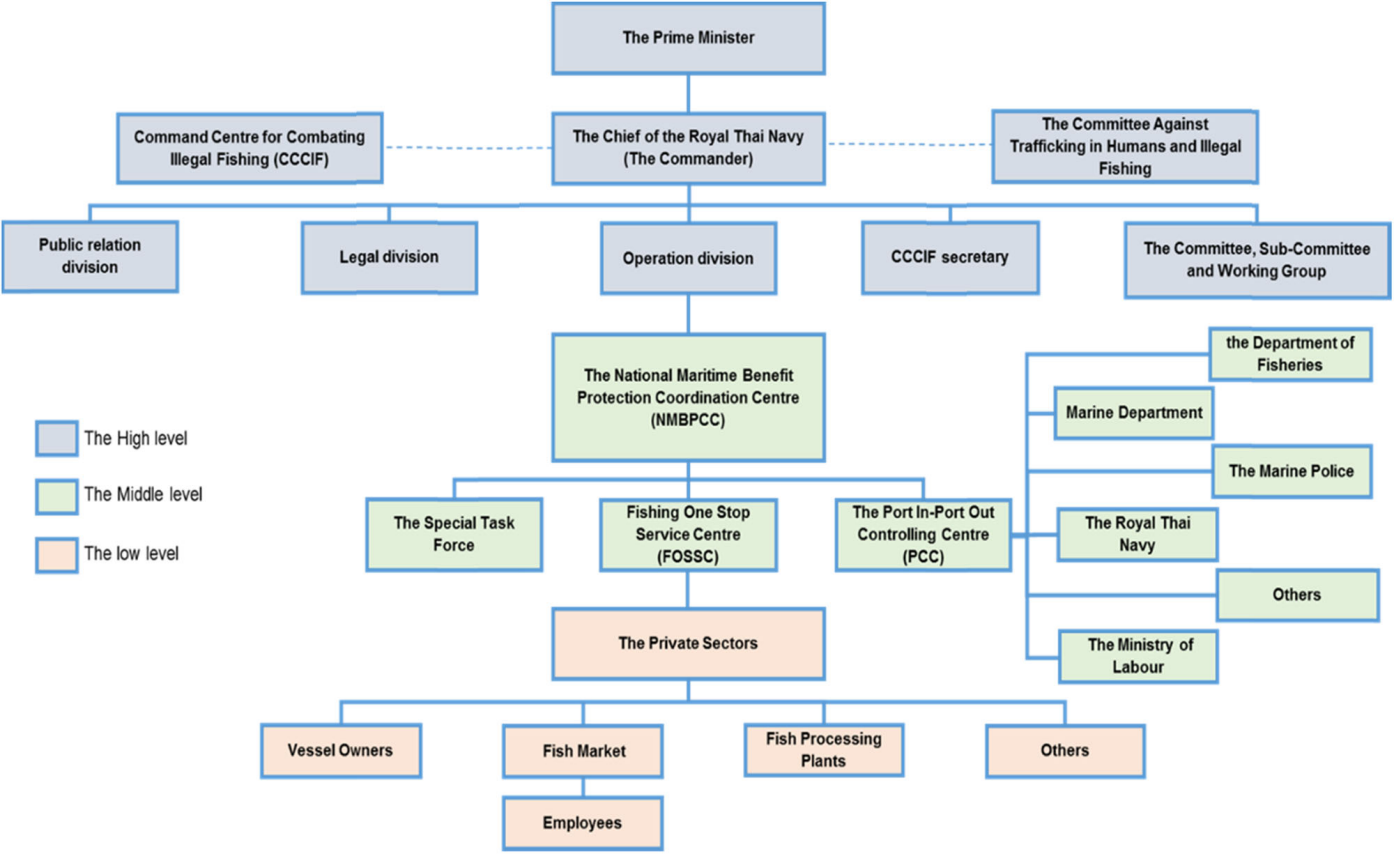
Simplified hierarchy of Thai fishery sector stakeholders directly in control over the management of IUU fishing

The regulations that try to control for the range of complexities could also encourage failure (Healey and Hennessey 1998), for example due to overly complex laws (see Agnew et al. 2009; Schmidt 2004). International fishing regulations are possibly the most pressing externally imposed constraints on global organizations’ strategic decisions (Elkington 1994; International Institute for Sustainable Development, Deloitte & Touche and Business Council for Sustainable Development 1992). While the Thai Government had taken steps to enforce these EU regulations—possibly for the combined reasons of IUU fishing and labor issues (Kadfak and Linke 2021)—to enact local laws and ensure local community compliance, the group of stakeholders that make up the Thai fishery sector is both broad and diverse. Therefore, they are difficult to enforce, given also a range of conflicting self-interests and constraints which were unknown to the EU Council at the time of enacting such regulations. Research has revealed a longstanding failure of local frontline fishers to follow regulations strictly, their continued lack of knowledge of rule compliance regarding gear usage, and even corrupt relationships between government officials in the Andaman coastal area (Bennett et al. 2014). Thus, the governance of human and labor rights is a complex dynamic that goes beyond simply environmental concerns (Wilhelm et al. 2020), and the full scope of stakeholders involved in the Thai fishery sector is too extensive to categorize accurately. Figure 1 thus outlines the direct line of command and control of key stakeholders most involved in combatting IUU fishing in Thailand concerning the present research, although in practice sub-committees also represent multi-stakeholder groups (see Kadfak and Linke 2021; Kadfak and Antonova 2021).

### Configuration Theories for Fisheries

A number of configuration theories and typologies exist for the grouping of organizations and various stakeholders for understanding their likely behavioral tendencies. A key problem is that the strategic thinking associated with such typologies was intended for narrow economic and business performance outcomes in mind, when a broader set of stakeholders are important in fisheries management (Haapasaari et al. 2013). Clarkson (1995) alternatively considered corporate social performance as the output, within whose theory incorporates a broad range of stakeholders—such as employees, customers, shareholders and suppliers. Drawing on former corporate social responsibility constructs from Carroll (1979), Clarkson’s blame of the internal manager is not for not achieving the outcomes of the organization but for not balancing the broad range of stakeholder needs. While Clarkson’s argument is important, environmental sustainability is not a constant social performance measure that can be adjusted intentionally, particularly as the vast number and diversity of stakeholders make the process dynamic (see Hauge et al. 2009).

In essence, a framework is necessary for understanding how different “organizational” (interpreted broadly as an organized entity), or stakeholder, types (i.e. with specific configurations) can respond to the various and specific external regulations to achieve congruity with the organization’s conventional practice (Miles et al. 1978). As such, the Miles and Snow (1978) typology is a framework that has been widely considered within the literatures of various industries (see Lin et al. 2014; Martins et al. 2014; Bordean et al. 2011; Borch 1999; Conant et al. 1990). One reason is because the typology enables stakeholders “to provide a parsimonious framework for describing complex organizational forms and for explaining outcomes” (Doty and Glick 1994, p. 230). The theory deals with the alternative ways organizations define their strategy, construct business structure and processes to pursue these strategies in responding to three core concerns: choice of a target market, the appropriate means to offer products and services, and the management of work. These are all relevant to broad organizational types (Zahra and Pearce 1990), and therefore other stakeholders, in the fishery sector.

The process of aligning organizational decisions with uncertain environments is complex, but can be eased whenever firms can describe and predict the adaptation of the processes through understanding the pattern in their behaviors. The Miles and Snow typology categorizes organizations by their patterns of decisions as three viable strategic archetypes—defenders, prospectors and analyzers—together with a non-viable archetype—reactors. Hence, the typology provides a description of organizational behaviors, key elements of strategy, structure, variable processes and the relationship of performance to the organizations (Witcher and Chau 2014). These enable stakeholders to build a distinctive competency (Snow and Hrebiniak 1980). For example, prospectors emphasize development of product and market effectiveness, and their distinctive competencies lie in product and research development and market research. On the contrary, there is no clear competence for reactors (Rajagopalan 1997). In essence, identifying and understanding the likely archetype into which stakeholders of the Thai fishery sector may facilitate understanding the response to any regulatory imposition and extent of impact.

Fishery stakeholders, not just business owners and fishers, might thus be attributed as follows. Prospector stakeholders will be most proactive in implementing new approaches relating to new market trends, and support new legislation in so doing. Innovation (not necessarily technological, but in the sense of using a range of approaches and procedures) in their overall outlook may be adopted without significant financial constraint. For example, officers whose work is to inspect legislative compliance are not affected by financial instabilities (Kadfak and Antonova 2021). Uncertain environments are challenging, so support of flexibility in the fishery approaches, such as different ports and changing fishing gear types, might be used, for (or if this applies to) commercial entities such as industrial-scale fishers. Wide communication and cooperation, because of their likely ability to do so, enable general efficiencies. Defender stakeholders seek stability by using limited fishing approaches throughout the year so they cannot spread risks. They also tend to use standard technology so their ability to respond to changing conditions is restrictive. They achieve cost efficiencies possibly through centralized control, minimal communication and extensive division of labor. Such stakeholders are likely to be commercial groupings that have been in operation for a long while (e.g. skipper and vessel businesses) that have established approaches for their activities and are less keen to adopt new practices. For example, research by Wilhelm et al. (2020) found that fishers generally lack confidence to raise issues either to authorities or take a leading role in innovations. Analyzer stakeholders exploit new opportunities and maintain their core fisheries simultaneously, thereby positioning between defenders and prospectors. They are dynamic and flexible, yet also stable—so there is likely to be an overlap with stakeholders of the defender archetype, but likely to include the smaller-scale fishers because their contribution to local communities is nonetheless significant (see FAO 2022). Such mariners might squeeze gains from fish prices while focusing on the quality. A matrix management structure is suited for administrations, and communication and cooperation are used significantly to achieve efficiencies—this is partly because of their involvement in value chains and post-harvest decision-making processes (FAO 2015). Reactor stakeholders may casually be seen as (business) failures because they are usually inconsistent and perpetually unstable in adjusting their strategies to their environment. They are financially risk-averse, so they are weak at managing costs. The quality of output/performance tends to be low due to poor communicational and cooperative skills. They are likely to be at the bottom of the value chain with restrictive power, but for the context of sustainability management they might not necessarily be weak in their value contribution. Smaller-scale fishers and local communities in fact make a significant contribution to environmental sustainability (Teh and Pauly 2018),

Miles and Snow (1978) contend that defenders, prospectors and analyzers can proactively respect their environment through unique proactive ways while reactors will only be responsive to most negative issues than take full advantage of any positive ones, suggesting the three proactive archetypes perform equally well, but outperform reactors. Their view has since been contested; for example, Zajac and Shortell (1989) argue that defenders fall significantly behind the other archetypes (see also Andrews et al. 2008a; Boyne and Walker 2004). In some cases, defenders outperform prospectors and reactors are not necessarily the worst performing configuration (Meier et al. 2007). As there is no evidence the typology will function in the same way across different industry environments (Hambrick 1983), Woodside et al. (1999) challenged the true linkage between the archetypes and performance outcomes. Thus, a limitation of the typology’s practical use, originating from a business usage in mind, is its strong reliance on single environments, which may prove problematic in areas of legal pluralism (see next section)—i.e. where the legal conditions may create more than one environment with which to determine archetypes for categorizing stakeholders. Hence, there is not an obvious archetype that is best capable of delivering regulatory objectives, which is further complicated by the extent (and intention) of stakeholders’ involvement in achieving these objectives. Another drawback of the typology is its inability to establish a causal link between constructs (e.g. regulatory and industry actors) to drive superior performance; in other words, a careful categorization of key stakeholders may provide invaluable insights about behaviors but will not provide the solid solution for environmental sustainability management.

### Managing Environmental Sustainability

A starting point for understanding the complexities of environmental regulation is to challenge the naïve view that all stakeholders are in favor of, will fully benefit from, and are therefore willing to engage in it. The so-called “Porter Hypothesis”, which asserts that well-designed environmental regulation will encourage stakeholders to innovate and lead to profitability (Porter and van der Linde 1995), is therefore flawed and weakly supported (Lanoie et al., 2011), in favor of the competing “costly regulation hypothesis” (Palmer et al. 1995) which takes into consideration cost constraints suffered by a range of stakeholders involved in the process (see Rassier and Earnhart 2015). Simply put, not all stakeholders will value the positive effect of stricter environmental control if high costs will be incurred at the individual level (Smith 1980). For example, if the application of high technology sustainable fishing may prolong fish stocks for the intermediate future the high cost incurred at the onset will outweigh any obvious benefits. This view is important to the Miles and Snow typology as the archetype into which the different stakeholders fall also depends on the level of innovation adoption for carrying out tasks, such as regulatory objectives. Despite the benefits outweighing the costs in the long-term (Horvathova 2012), those stakeholders most likely affected are those least able to afford the immediate cost incurrence such as small fisheries. Another issue is that of trust—how knowledge flows between decision makers (legislators/regulators), scientists (about the benefits of environmental control) and the stakeholders (fishers who deliver the objectives)—within the governance system (Holm and Soma 2016). Principles of effective environmental regulation dictate that approaches and governance mechanisms need, inter alia: to be participative, to induce innovation, and to be supported by adequate resources (de Miranda Ribeiro and Kruglianskas 2015). The interactions between resources, the governance system and affected users (fishers) are closely tied (Ostrom 2009, 2010).

Satumanatpan et al. (2017) argued that the failures of engaging stakeholders in local policies are caused by a misunderstanding of the governance system and alignment to local needs. Between stakeholders, local fisheries legislations are poorly implemented because of the lack of coordination and integration of the core processes involved (Khumsri et al. 2009). Engagement in the legislation was hindered by the absence of capacity building among the local community. Resilience in any such governance systems requires the explicit participation of local communities (Jones et al. 2013). Research by Bennett and Dearden (2014) on the perceptions of conservation management in the Andaman Coast of Thailand has recognized local fishers’ fear of increased poverty, food security, wellbeing and access to fishing as significant barriers to any environmental program. They argued that improvement concerns “the broader array of issues, and their root causes are taken into account and that management actions are coordinated between agencies and across the Andaman coastal zone [and] … processes and outcomes will also necessitate partnerships with organizations that are better equipped to address development issues … to cast a much broader net – to be amenable to coordinating with other governmental and non-governmental organizations and to including local communities …” (ibid, p.115). Similarly, Hines et al. (2005) found that sustainability in the Andaman Coast requires sound integration of governmental and non-governmental institutions as well as local communities. These views are in support of findings by López-Gamero et al. (2010), that command-and-control legislative approaches are less effective than those which stem from voluntary norms. Hence, there is a significant disconnection between externally imposed targets set by regulators and the specific stakeholder motivations for achieving them. It is not necessarily the case that the stakeholders do not perceive the benefits of controlling specific marine protected areas, but that they understand them better when they relate them to their own activities and perceptions (Leleu et al. 2012). So understanding the perceptions and behavioral intentions of the various stakeholders provides a good indication of the likely success of the outcomes.

The closest form of connecting a country’s industry stakeholders with external standards may be an adaptive form of co-management system (where legal pluralism—the application of multiple legal systems for the same area—might exist), which has become a popular and preferred institutional arrangement for managing fisheries (Chuenpagdee and Song 2012). “The state is not the only legislator; law is not unique to state societies; and other forms of law exist … Further, civil society is not a passive recipient of rules and regulations; rather, different sectors are often involved in shaping rules … [L]egal pluralism is premised on exploring legal and political configurations without any bias toward a particular source of law” (Parlee and Wiber 2014, p.48). Developed and operated effectively in Europe, it argues that sustainability planning must enable social and institutional components for effective fisheries co-governance. However, much is still unknown about “fisheries co-management in trans-sectorial coordination and in networked and globally managed resource use is also still a necessary component of attempts to address uncertainties and risks at various levels of resource management” (Linke and Bruckmeier 2015, p.180). Bavinck and Gupta (2014) outlined four types of pluralist governance systems: (1) indifference, that lacks operational overlap between fields of interest, (2) competition, where tensions exist between legal systems and governance patterns, (3) accommodation, recognizing reciprocal adaptation but with poor integration, and (4) mutual support, of true partnership between all stakeholders and local and external regulatory systems. While it is clear that the last type is most positive and desirable of the whole Thai fisheries context, we believe these characterize the Miles and Snow configurational typology and throw light on understanding individual motivations. With their respective typological solutions—such as (1) knowledge and capacity building, (2) negotiation and conflict resolution, (3) co-management of resources and mutual learning, and (4) improvement of routines—these specific strategies would lead to better delivery of the environmental objectives if directed to the specific stakeholders of the Miles and Snow archetype.

For example, we believe (1) indifference relates to reactors because of their inconsistent and mismatch of issues. By that, if stakeholders are unable to respond to manage policy objectives effectively—but occasionally does manage to do so, there is an indifference in the outcome, and reactors continue to operate in the same way. Contrarily, (2) competition relates to defenders who are stakeholders who stick to familiar and internal conditions with a low willingness to engage. In other words, they defend the status quo by making newly desired outcomes difficult and operate in established ways, creating a competitive environment for them to be achieved. This may be most prominent in indigenous and local communities with long-established fishing rights. However, (3) accommodation relates to analyzer stakeholders who exercise either or a number of approaches and can see and benefit from compliance with new requirements. These may be commercial firms whose interests might be broad ranging, and are open to deviating from practices that might be to their or others’ advantages. Lastly, (4) mutual support relates to prospector stakeholders that are proactive in adopting new innovations. This might be because there is a direct obligatory demand for immediate implementation (for authorities) or they see harmony and benefits to the majority of stakeholders if compliance is achieved.

If our prima facie thinking is correct, prospector stakeholders might be assumed the most effective at delivering sustainability objectives, in support of legal pluralism ideals. However, Maniora (2018) recently compared prospector to defender archetypes, and argued that prospectors are more intentionally likely to *mis*manage sustainability objectives than defenders because of a difference between material and immaterial sustainability issues—such that material issues cost more and immaterial issues are carried out mainly on a reputational basis. In other words, mutual support is purely lip service application! Her view is that prospectors have rapid sporadic growth patterns and will fail in engaging in the right material sustainability issues (both unintentionally and intentionally, but intentionally overall)—in other words, they will *mis*manage. Defenders instead, she ascertains, are simpler and better co-ordinated and will therefore engage in the effective management of sustainability issues (despite a general reluctance to engage overall). Maniora’s (2018) classification of archetypes into intentionality and mis/management of sustainability issues is similar to the argument of Schaltegger and Burritt (2018). Flaws and problems were rightly raised about the reliance on existing tools for justifying the business cases for sustainability involvement (cf. Salzmann et al. 2005). Thus, Schaltegger and Burritt suggested there are different motivations for businesses in engaging in sustainability issues, distinguishing the “business case of sustainability” (because firms need to for reputational and reactionary reasons) from the “business case for sustainability” (because there is either a collaborative effort to engage or an ethical responsibility to do so) but neither guarantees their success (or perhaps better put, the successful material management of sustainability issues, borrowing the lexicon and nomenclature of Maniora).

The application of the Miles and Snow typology in the Thai fishery sector might enable stakeholders to understand strategic behaviors that enable efficient adaptation to the external change and uncertain conditions. Externally imposed constraints, such as the specific EU IUU fishing regulations, constitute the external environment that fisheries’ strategic choices and the competitive strategies are constrained in sustaining competitive advantage, and reduction of chances of misalignment as well as bring sustainable benefits to the industry. These are best understood in conjunction with the motivation frames of Schaltegger and Burritt (2018) and the mis/management of sustainability issues categorization of Maniora (2018), as presented later in the present research.

## Methodology

The research involved first-hand exploration of key stakeholders in the Thai fishery sector to understand how they are constrained by the contextual circumstances when complying with the specific IUU fishing regulatory objectives.

### Research Design

Key stakeholders of the fishery sector in Thailand located in the Satun Province of the southern Andaman Coastal region were selected for the present study, as this is an important, high yielding fishing area, and historically had broken off as a Malay State and formerly connected to the British Empire. It is located close to three national parks of protection, and has a strong provincial governmental make-up. The stakeholders included individual fishers and personnel of institutions of various levels of authority in the sector, which were already shown in Fig. 1, to ensure comprehensive coverage. This represents a “case”, from a methodological perspective that allows extensive understanding of real life phenomena (see Yin 2018), in which the physical boundaries are that of the fishing area. However, the highly connected instances of being a longstanding community establishment reliant on fishing (Panjarat 2008), and which has had fishing practices constrained by IUU fishing control (EJF 2019), offer a good opportunity for respondents to give opinions that are exemplary of regulatory constraint.

Operating in strict accordance with the approved procedures of the researchers’ institutional ethical approval board for the project, an interview guide was designed and presented to all the stakeholders (with small variations to reflect their specific role and context) based on characteristics that define the Miles and Snow typology and features of IUU fishing drawn from the specific EU Directive. Hence, the interviews were in-depth, semi-structured and open-ended to allow the probing of context-rich and self-reflective commentary. This meant that we could understand how a specific theoretical framework would apply to the specific fisheries context. The issues examined focused on both the positive aspects of the work as well as the constraints of IUU fishing regulations. This is not to say that all data collected relied on the interview commentary; as extension of a theoretical framework into a specific context is seen as a middle range methodology (see Laughlin 1995) we had some prior knowledge of the fishery constraints, based on the extant literature, which acted as a prompt for further probing for the specific research context and facts for verification or exploration of insights that are not within the extant literature.

### Data Collection

Rich commentaries were collected from in-person face-to-face interviews with key personnel (i.e. those who had a controlling responsibility to executive vital activities of their business) within the fishery sector in the Satun province. The respondents were therefore knowledgeable, as they were affected by the regulatory issues of concern, and therefore they were deemed appropriate participants (from a methodological perspective), to comment on the issues of research interest. The segment included primarily the PIPO (port-in port-out) Control Center, the fish market and the processing plant, which are core to the fishery sector and most directly impacted by any IUU fishing constraints. A total of 13 interviews were conducted, which comprised 8 participants from the private sector and 5 participants from the public sector. A summary of the interviewee profiles is given at Appendix A. The sample size was deemed adequate because the key issues of research were already provided by the majority of the respondents, and were overlapped by a range of stakeholder groups, which minimized the existence of bias. This criterion was deemed methodological sufficient as the participants were core to the issues of research and heterogeneously representative (Kuzel 1992; Saunders and Townsend 2016), and theoretical saturation (a phenomenon deemed important for case research) was already observed during the data analysis process, where the researchers felt “no additional data are being found … can develop properties of the category” (Glaser and Strauss 1967, p. 65). The involvement of these two sectors enabled understanding of the disconnect between public organizations that have associations with governmental departments involved in implementing IUU fishing regulations—such as the Marine Department, Fisheries Department, Marine Police and Department of Labor Protection and Welfare—and the smaller fishing stakeholders in the private sector that had no involvement in the legal and policy making aspects of fishing. Data collection took place over a 3-month period, between June–August 2018, which was a critical period in which Thailand’s yellow card was still in effect.

Access to the first few key respondents was obtained through making direct contact with a senior officer of the fishery sector who found interest and felt it beneficial to engage in the present research project, who arranged the meetings, but was not directly involved in the research (interviews) to avoid conflict of interest or any form of persuasion that could have biased the results. Sampling was the outcome of snowballing, after successful agreement/completion of one interview with one respondent leading to the introduction of another interviewee, as part of a networked group of actors within the local fishery sector. Each new respondent was by agreement with the research team to ensure suitability, to prevent bias, and was not the complete decision of the most recently interviewed. The mix of respondents was diverse, representing mainly middle and low levels of the fishery structure, and ranged from 3 to 20 years of fishery experience. These levels of authority in the sector offered a suitable and representative grouping of respondents and institutions as they were all affected in some way by the EU IUU fishing regulations, being either most constrained as a consequence of compliance or most stretched in implementing them.

The interviews covered broadly three parts: general background information about the respondent and work (to gauge the extent of the stakeholder impact), specific EU IUU fishing regulation’s impact on work and compliance (for the technical context of application), and specific respondent views on the sustainability of the Thai fishery sector and general recommendations (to understand the emotional opening up of the individuals). All respondents remained anonymous and could not be individually identified from their profile descriptions after the interviews, and the commercial companies were subsequently pseudonymized. The verbal language of communication used was Thai, particularly the local dialect, to facilitate comfortability of the interviewee; this was enabled by one of the members of the researcher group being fluent in the language and was knowledgeable of local people in the sector. The use of such a skilled interviewer most likely had helped gain more representative commentary (Krefting 1991). Interactions mostly lasted over an hour in duration, which were typical of Thai hospitality and sufficient time to cover the core issues of investigative interest. They were digitally recorded and initially transcribed verbatim in Thai and then translated into English, using the standard back-method (Breslin 1976) to preserve the true meaning of the conversation; this was carried out by the same person to avoid confusion with use of terminology, which hopefully led to a more consistent analysis of the data.

### Data Analysis

**Fig. 2:**
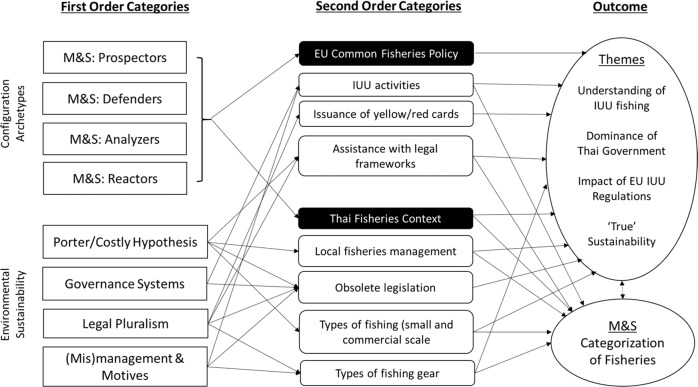
Template analysis for deriving themes from the research

**Fig. 3:**
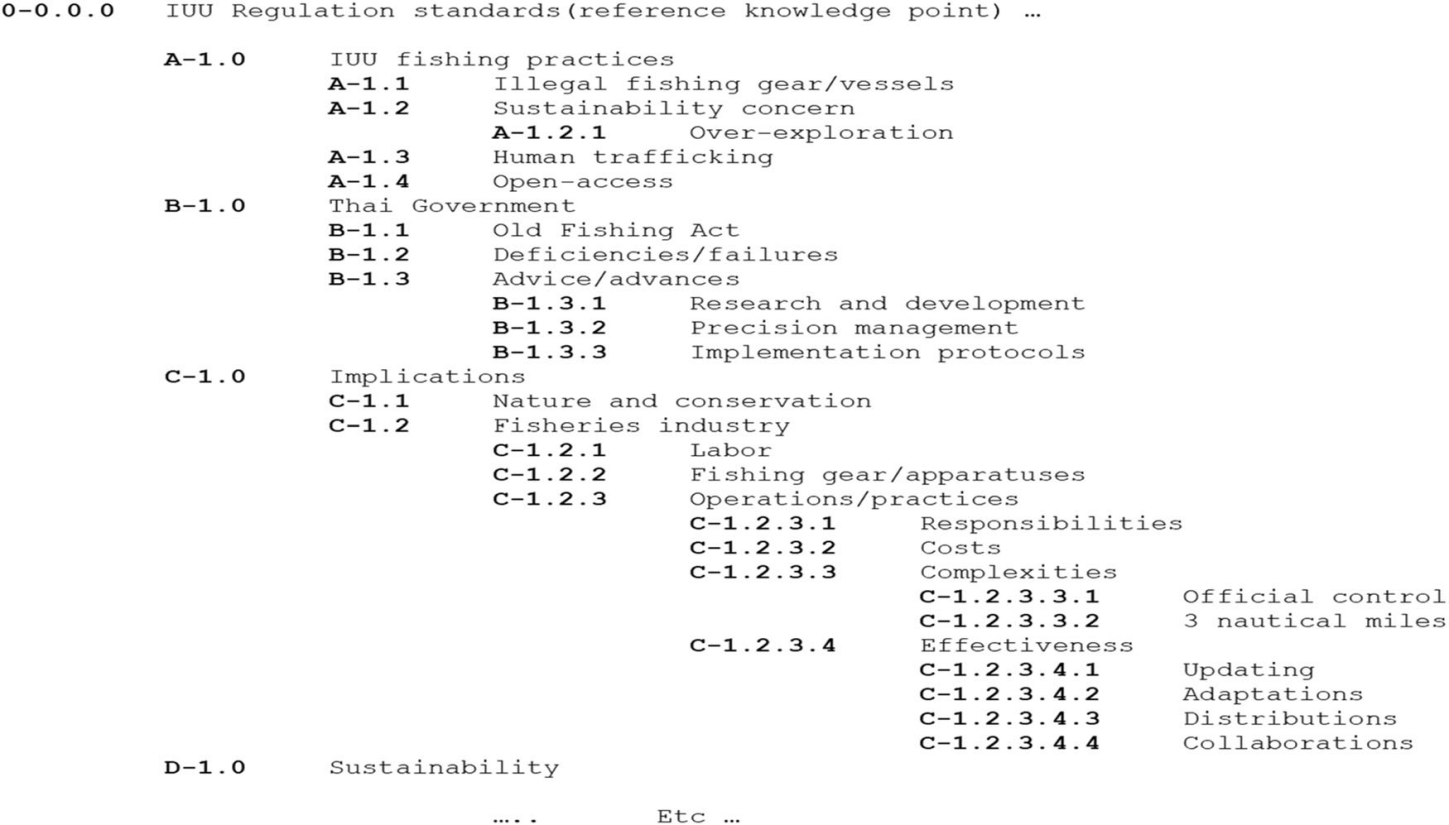
Coding tree (condensed partial illustration)

Data analysis involved applying a version of thematic qualitative data analysis, which was adapted from King’s (2004) template analysis technique. The idea and suitability of this technique are that a prior template of anticipated groupings/application (such as the Miles and Snow archetypes) would prompt data extraction from the empirical context, then fleshed out by additional prompts of the interested issues (IUU fishing regulations), to enable both consistency of the template and robustness of exploration. A simplified five-staged procedure was operated, as follows:

1. Detailed transcription of interviews: the original interview commentary was transcribed into text in the original language (Thai) verbatim, and then translated by the same researcher into English. This was then transposed onto a specific layout format involving large margins to allow for subsequent manual coding and reflexive appreciation (stages 3 and 5 below).
2. Establishment of a priori themes: a prior set of categories relating to the typology and sustainability concepts/literature formed a skeletal framework with which to guide the first-hand investigation as the diverse stakeholders discussed a range of topics that required refocusing. Hence, the interview information relating to strategic postures of the typology formed the first-order categories, their connection to any form of IUU fishing standards or Thai local fishing as the second-order categories, and patterns of themes that emerged became the third/final-order categories (see Fig. 2).
3. Development of a coding system: “In vivo coding” (Strauss and Corbin 1998) was used to code the interview data. This was carried out through the allocation of the higher order (called “parent”) codes, with the lower order information (“child”) codes, using alpha-numerical labeling. Figure 3 provides a condensed illustration of the coding tree used. For example, B-1.3.3 represented the implementation protocols necessary for the Thai government to implement legislation. This was carried out iteratively as the interviews progressed to ensure a constant referring back to the theoretical framework.
4. Identifying clusters as patterns of emergence: patterns of themes were identified using a simple form of content analysis. The most common and frequently recurring words/phrases relating to the issues (or variants of them) of the skeletal framework (as described in stage 2 above) were frequency counted and tallied to identify where clusters appeared. The words/phrases were not weighted when identifying the clusters as doing so might pre-judge the importance of the issues raised by the interviewees, which allowed both manifest and latent meanings (Berg 2001) to emerge.
5. Reflexive evaluation to ensure research quality: as many of the hallmarks of the qualitative methodological approach employed were capitalized using minimal people within the research team—such as the same person for interviewing, transcribing, translating and data analysis—there also ran the risk of bias/subjectivity and credibility in the event of human error. Thus, a set of manually written notes for the whole duration of the field study and data analysis was kept. Only two small issues had been identified. First, one of the researchers had a relative who knew one of the interviewees, but this was not known to the interviewee which meant it was too remote and unlikely to have influenced any response. Second, the same researcher had significant knowledge of the difficulties one of the fishers was opening up about, so there was a small possibility of probing excessively on a specific pre-judged issue during the interview. On balance, it was felt the need to explore core issues relating to the empirical context outweighed the likelihood of the existence of bias.

## Findings and Empirical Contributions

The prominent themes that emerged from the empirical research, are as follows. The connection of the claims to the data are given by the codes in square parentheses [].

### Understanding of IUU Fishing

It seems the interviewees had moderate understanding of what IUU fishing regulations generally concerned and how important they were for sustainability, and such understanding was paramount in a general agreement with them. This was evidenced by the most common comments relating to IUU fishing (Interviewees 1, 2, 4, 6, 10 and 11) and fishing without any sustainable concerns (Interviewee 3, 5, 7, 12 and 13)—[codes A-1.1, A-1.2]. The composition of IUU fishing was highlighted as important (Interviewees 1, 3 and 13); these included fishing vessels, fishing gear, and labor. However, some participants were confused between IUU fishing in general and the specific EU IUU fishing regulations; for example, it was commented:

*“IUU fishing is about legal fishing which comprehensively controls the whole fishing industry and includes labor.”* (Interviewee 9)

This suggests their reference to the local controls (unsuccessful legislation, deemed problematic by the EU for achieving sustainability targets) rather than the new EU compliances. As the latter is more developed and tougher, any misunderstanding would inevitably reduce any possibility of their success even if there is a general positive attitude toward a sustainable future.

Applying illegal vessels and fishing gear was prioritized as the primary cause of IUU fishing (Interviewees 1, 5, 9, 10, 11, 12 and 13), followed by human trafficking (Interviewee 4, 10 and 12) [code A-1.3]. These two reasons commonly take place in many countries, and Thailand is no exception when fishing represents a significant livelihood. Specifically, Interviewees 7 and 8 believed the essential cause of IUU fishing in Thailand is the application of an extended open-access fishing system [code A-1.4]. Hence:

*“Previously, the Thai fishing sector used an open-access system which seamen and fishers independently operated, and fishing documents were never controlled.”* (Interviewee 8)

A consequent effect of applying destructive fishing equipment and catching fish without further concerns leads to an over-exploration [code A-1.2.1] which directly decreases marine resources (Interviewees 1, 2 and 5). Nevertheless, it seems:

*“The main reason for the decline of marine resources does not primarily come from IUU fishing, but because of global warming. Nowadays, the sea temperature is approximately 34–35 degrees Celsius, compared to the last twenty years, which was around 29 degrees Celsius. As a result, spawn and juvenile fish cannot live.”* (Interviewee 11)

Moreover, a dual nationality vessel such as Thai/Malaysian was believed to be another cause of IUU fishing in Thailand (Interviewee 13), meaning that there may exist legal loopholes which need to be closed to be able to address the practical concerns.

The broad range of stakeholders have concurred a close connection with the fine details of IUU fishing, which suggests a significant impact on the whole sector. The broad reasons of blame given therefore evidence the need for more individualized mechanisms within any IUU fishing legislation to be able to target and maximize stakeholder compliance.

### Dominance of the Thai Government

The Thai government is the key authority in eliminating, preventing and deterring IUU fishing in Thailand, which might be regarded as a positive influence on ensuring effective management of sustainability issues. In this instance however government was also regarded as a negative force rather than a supporting condition [code B-1.2]. The interviewees believed the government had not placed sufficient effort in solving the associated problems in a practical way. Some of the interviewees explicitly criticized the Thai government’s efforts as deficient and unprofessional, leading to increased opportunities to engage in IUU fishing. The criticisms initially started with comments on the inefficiency of the old Fisheries Act (B.E. 2490) which had been used for nearly 70 years [code B-1.1], followed by ineffective setting of new local legislation—the Royal Ordinance on Fisheries B.E. 2558 (2015)—to align with the EU fishing standards. Interviewees 2, 3, 4 and 6 pointed out that the former legislation itself was the key cause of IUU fishing because Thailand’s rule of law was too weak to start with. And while new fishing regulations were implemented, compliance with them was poor among the stakeholders (Interviewee 8).

Other reasons demonstrate the inefficiency of the Thai government’s performance [code B-1.3.3]. For example, controls over the fishing sector were not seen as rigorous enough and offenders are still roaming at large (Interviewees 4, 7, 8 and 9), and that the government lacked the sufficient workforce and budget to support the implementation plans (Interviewees 2 and 3). Additionally, one interviewee pertained that the government has not considered the stakeholders of IUU fishing proportionately:

*“The most important cause of IUU fishing comes from a pair trawl net which is powerfully destructive, and yet the government applies the IUU fishing regulations equally with everyone.”* (Interviewee 10)

On asking interviewees to score the effectiveness of the government’s effectiveness in combating IUU fishing (where 100% denotes most effective), the average was 64%. Only Interviewee 4 (unsurprisingly a legal officer) scored them 100% because of a legal commitment to the Thai government. On the contrary, efficiency was scored 0% by Interviewee 6 (a plant owner), who explained:

*“I have never heard that the Thai government is attempting to negotiate with the EU Commission. They only perform to follow the EU. Thus, I do not believe in them. Even though illegal vessels have been arrested and stopped operating, why is there still an overfishing issue in Thailand?”* (Interviewee 6)

Uneducated officers are another reason believed to cause IUU fishing:

*“There is no effective training program for the government officers and, I am more knowledgeable of the updated Fishing Act than the officers. There is no consistency; state officers at each PIPO Control Center operate differently.”* (Interviewee 6)

However, the interviewees made helpful recommendations for the Thai government on the enhancement of efficiency, namely, that more consultation with fishers is necessary before implementing new requirements of external standards, or even managing pre-existing laws. One regulation may not perfectly be applied to every type of fishing gear and vessel, and so doing more empirical research may enable understanding of the benefits and drawbacks [code B-1.3.1] as well as practical approaches of each kind of fishing gear (Interviewees 6, 7, 11 and 13). Alternatively, the implementation plan should be more considered and have a clear agenda such as a re-enrollment date for migrant workers and registration days (Interviewees 6, 7 and 9). Moreover, following the EU more broadly in their other sustainability policies may be another way to increase efficiency because there is greater confidence in the EU’s capability to eliminate IUU fishing (Interviewees 1 and 4):

*“I do believe that the EU Commission has strong potential for eliminating IUU fishing; not only in Thailand, but we can see in many other countries such as Brazil. Therefore, Thailand should imitate the EU entirely.”* (Interviewee 4)

Other interesting advice even included the request that government pay more attention to local/small businesses that lack financial resources and legislators come from the regional sector instead of appointing academics for the role who have never approached local fishers (Interviewees 2 and 9). Use of educational training programs might also encourage government officers to be more professional (Interviewee 13).

Overall, the role of government, while often considered ineffective by the stakeholders, is a dominant one in Thailand. Its legal position holds the key to the specific regulations’ success, and that strength of influence should be harnessed carefully.

### Impact of the Specific EU IUU Fishing Regulations

The implementation of the IUU fishing regulations has generated both positive and adverse effects on the environment and the Thai fishery sector. More than half of the interviewees believed that the fishing regulations would lead to a significant improvement to wildlife and environmental conservation, and consequently, marine resources would gradually become sustainable over time [code C-1.1]. The reason for the growth in natural resources is believed to be due to the reduced operation of illegal fishing gear and illegal vessels (Interviewees 2, 5 and 12).

Similarly, labor forces seem to be one of the essential compositions of the Thai fishery sector [code C-1.2.1]. Under the control of the EU IUU fishing regulations, migrant workers (normally the most problematic of illegal activities) seem more organized and easier to control (Interviewees 7, 8, 9, 11 and 12). For example, it was noted:

*“It is [now] much easier for me to control my underlings. Formerly, there was no official supervision over migrant workers, but, nowadays, migrant workers must sign an employment contract covering a one-year operation. Hence, their turnover rate is slightly declining, and our relationship is getting closer.”* (Interviewee 12)

Nevertheless, the regulations have not always been positive, and in some circumstances can be difficult to manage. For example:

*“The [IUU fishing] regulations prohibit over 55-year old migrant workers, excluding the ones who have a passport, working in any fishery organizations. Even if they still have the strength and capability to work, we cannot employ them. Also, getting passports is costly for migrant workers. Therefore, we have to recruit new workers – who are less experienced – to fulfill the vacant positions.”* (Interviewee 9)

In terms of fishing gear, its volume, such as pair trawl nets, has likely decreased significantly after implementing the fishing regulations (Interviewees 2, 5, and 12). This possibly resulted from the regulation that categorized gear as illegal fishing that is known for destroying natural resources [code C-1.2.2]. Unfortunately, it seems to produce some adverse effects on fishers who have possessed that gear before the classification. As explained:

*“Many types of fishing gear have been available in Thailand for a long time. Once the new regulation was implemented, a pair trawl net was legally classified as illegal fishing gear. So, there is a negative impact on fishers who have used the equipment before the implementation. They had to stop sailing permanently and close their business. I think it is unfair for them.”* (Interviewee 8)

Moreover, there were complaints about the implications of implementing the IUU fishing regulations on fishers’ careers, particularly in relation to the increase in work responsibilities (Interviewees 1, 2, 3, 4, 7, 8 and 9). This was also the case for government officers who were appointed as operating officers at PIPO Control Centers, thereby increasing costs of operations [code C-1.2.3.2] and the setting up of new machinery (Interviewees 1, 4, 6, 7 and 11).

Interviewees also commented on the complexities of working processes [code C-1.2.3.3]. The Thai fishery sector had never hitherto been controlled intensively. After becoming officially controlled by the government, operating procedures got complicated. For example, various official documents, such as processing statements, fishing logbooks, and employment contracts for outlanders, were legally required by the government to trace and supervise workers efficiently (Interviewees 4, 7, 8, 9 and 10). However, it was a significant problem for most migrant workers operating at sea who were uneducated or illiterate. Some other rules have already affected fishers’ operational effectiveness [code C-1.2.3.3.1]:

*“The new regulations limit the number of migrant workers on board and in some positions; for example, a repairer must be Thai. Even if migrant workers have more professional skills, we cannot employ them. For this reason, the overall performance is not effective as it should be.”* (Interviewee 12)

Similarly, a limitation of three nautical miles prohibits vessels exceeding 30 tons from fishing within three miles of the coastlines [code C-1.2.3.3.2]. This led to the scarcity of some species of fish such as anchovies being served to the market.

*“Anchovies naturally live near the seacoast which is within three nautical miles. Thus, anchovy fishers are forbidden to catch in the area.”* (Interviewee 2)

One interviewee who runs a business related to anchovies was significantly affected by the limitation of catch zones, as he explained:

*“A year after implementation, I am obliged to stop operating my business temporarily. Although I do not operate fishing gear and machinery, I have to spend an enormous amount of money on repairing them. It does not affect only me – my workers suffer too; around 600 laborers have become unemployed.”* (Interviewee 6)

Recommendations on increasing the efficiency of working performances were suggested by Interviewees 1, 2, 3, 5, 6 and 7, which included the need to update the latest Fishing Act and government’s announcements regularly, and then to adapt the working processes to the changes (Interviewee 5, 7, 8 and 11). While entrepreneurs might be patient with the EU IUU fishing regulations and the corresponding local legislation in protecting their business, they might not be happy with the extent of micromanagement.

*“The law forces all workers who work at the fish market to show their employee ID clearly all the time during operations. I know it is a tiny problem, but it makes them annoyed. If the regulations have more conditions which generate inconvenience in the workplace, the employees might eventually resign.”* (Interviewee 8)

These implications extend beyond the fishery sector if the fishing industry collapses (Interviewees 7 and 11), fearing the knock-on impact would create social and economic problems to the community offset by the high unemployment rate (Interviewees 5 and 10), whose livelihoods were already dependent on what the fisheries could provide.

### “True” Sustainability

The perception that the concept of (environmental) sustainability was rather abstract in practice emerged. Some interviewees did not believe sustainability was a “true” concept in that either it was either not taken seriously by the authorities’ approach in trying to achieve it or that it is a position that cannot in practice ever be achieved. Sustainability featured prominently in 11 of the 13 interviewee conversations, generally agreeing the fishery sector will potentially become more sustainable in the long-term [code D-1.1]. However, this is conditional of government regulating practically and efficiently and stakeholders abide by the regulations, and further that legal officers and the private sector work together collaboratively. However, the likelihood of these conditions happening was regarded low and difficult to achieve, resulting perhaps in a lip-service approach in achieving sustainability objectives. Likewise:

*“The IUU fishing regulations have been implemented to keep a balance of marine resources based on the calculation of the maximum sustainable yield (MSY). If the new fishing legislation genuinely controls the levels of catch under the MSY, then the Thai fishery sector will operate sustainably.”* (Interviewee 3)

In this sense, the interviewee (an officer) highlighted the case for how IUU regulations would theoretically control levels of catch. However, the effectiveness of legislation was doubted, when a different interviewee (a kipper captain) outlined the practicality of the regulations being followed:

*“The sector and its related businesses are possibly getting worse, because it is not easy to adapt working practices to every single regulation and entrepreneurs probably cannot afford the higher costs. So [‘true’] sustainability is not achieved.”* (Interviewee 11)

The credibility of the claim of local fishing sustainability was also however put into disrepute when Interviewee 6 observed a negative trend; Thailand was once the top five of the world’s seafood producers but is now importing fish that were historically ample in Thailand.

While the word “sustainability” may have come into use explicitly by the interviewees or by implication through discussion, its meaning and true application have varied. That is—a truly sustainable position for local businesses (by engaging in high volume fishing with low-cost practices) is necessary to support livelihoods, in practice contradicts with the environmental sustainability position that requires high cost investment into the future. If the urgency and temptation are for the former, coupled with ineffective governmental attempts to achieve the latter, then “true” sustainability cannot be achieved.

## Discussion and Model of Archetypes and Sustainability Motivations

The themes from the above findings were grouped within the four Miles and Snow archetypes and discussed further for how different stakeholders can best respond to the regulatory constraints. Table 1 combines the theoretical configurations. These discussions were then used to present a composite model for understanding how sustainability might be (mis)managed.

**Table 1:** Relationship of configuration theories and Thai fisheries stakeholders

Miles and Snow (1978)	Bavinck and Gupta (2014)	Schaltegger and Burritt (2018)	Maniora (2018)	Thai fisheries stakeholders
Prospectors—proactively seek opportunities and engage in innovation with minimal financial constraints	Mutual support—legal officers most able to support new obligations	Business case FOR (collaborative), Business case OF (reputational)	MISmanagement (due to being costly)	Department of Fisheries; Marine Department; Department of Labor Protection & Welfare
Defenders—seek limited approaches and standard technologies	Competition—cost burdens mean stakeholders compete against conservation objectives by non-investment in innovation	Business case FOR (responsible), Business case OF (reactionary)	Effective management (due to simplicity and coordination)	Fishing Company; Skipper Company
Analyzers—explore new opportunities while sustaining a stable position	Accommodation—occasional support of new obligations for those financially stronger	Business case FOR (responsible), Business case OF (reputational)	MISmanagement (due to being costly for being small player) or effective management (due to high adaptability)	Vessel Company Fishing Company
Reactors—perpetually unstable and financially weak and risk averse	Indifference—business reliance on other parts of supply chain that complies	Business case FOR (no business gain), Business case OF (reactionary and reputational)	Effective management (due to a difficult industry-imposed position that is the result of sustainability compliance)	Anchovy processing plant Fish market

### Prospectors

One might initiatively assume prospectors are risk taking fishers desiring to explore new locations for fishing opportunities. While this might make sense, Thai fishers lack the financial resources to adopt the necessary innovation to do so alongside the new IUU fishing regulatory constraints. Instead, only stakeholders with legal obligations to enforce the IUU standards (be it their true personal belief and/or interest in environmental conservation or not) without cost considerations comfortably fit this archetype. Our findings suggest government officers (Interviewees 1, 2, 3, 4 and 13)—consistent with Bavinck and Gupta’s (2014) categorization in applying a high level of mutual support—are most likely to be prospectors because they are strategically involved in the adaptations of the working processes of the updated laws. The regulations for combating IUU fishing are first legislated, and updates of their progress are reviewed by the regulators and officials who are positioned at high levels of the Thai authorities almost on a monthly basis. Thus, it is the responsibility of the middle level officers to modify their working procedures to match the changes of the regulations. The officers are in the middle between the regulators and the fishers; therefore they have to ensure that they are able to cascade the appropriate messages well to the lower level. Moreover, working efficiently in the entirely new environment and with colleagues at PIPO Control Centers intensely increases the ability to adapt themselves quickly to the new surroundings. Collaborating well might help to find the best possible way to perform effectively among different departments. Officers may not (and do not require to) have much personal input as part of their daily work as they are duty bound to follow legal orders. However, in practice they still adjust themselves to the current context, even though the environment surrounding them has entirely changed. For example:

*“I have to coordinate with other departments, such as the Department of Fisheries, which I have never corresponded with before.”* (Interviewee 2)

The officer’s comment does however suggest a lack of experience in coordinating with other officers due to the broader involvement of stakeholders (other legal officers) relating to IUU fishing. Nonetheless, the proactive approach in extending to legal entities is consistent with the view that public institutions are characterized as innovative organizations (see Andrews et al. 2005), adopting a prospector stance to search for new market opportunities and to experiment with potential responses to an emerging environment.

As prospectors are innovators who invest heavily in searching for new technologies to enhance efficiency, operations and collaboration through a network system possibly assist government officers to perform effectively. It probably means that innovation technology will help to search for new and innovative ways to provide the best service to their customers, comprised mostly of the migrant workers at sea who have little authority on fishing decisions. Additionally, innovation processes can improve the involvement of different departments (Fortuin et al. 2007). However, it is crucial for prospector stakeholders to choose the right innovation that suits the genuine organizational practices before they turn cost-inefficient. Should that happen, there might be clashes of priorities and the creation of vested self-interested objectives, leading to the lip-servicing and mismanagement of the sustainability issues.

### Defenders

Defenders are adept at processing and accommodating new conditions as a consequence of updated local legislation and compliance requirements, and frequently ensure the processes are suitable for the existing practices to achieve stability (Andrews et al. 2008b). They expend significant time in planning to enhance effectiveness and reduce costs. Private sector stakeholders (e.g. Interviewee 8, 9, 10, 11 and 12) are likely defenders. As the local restriction—the Thai Fishing License, valid for 2 years—only allows fishers and entrepreneurs to possess one type of fishing gear, and can operate only within one fishing area in the Gulf of Thailand or the Andaman Sea (Department of European Affairs 2016b), meaning some operational constraints do exist. So, they achieve cost-efficiencies by using single core technologies, such as fishers who apply a single gear to fish in the same locations, which suggests the imperative to engage in sustainability issues are better/more effectively managed.

The operatives (Interviewees 8, 9 and 12) in the skipper business also typicalize defenders because they are followers of strict company rules, which had set the rules to achieve cost efficiencies necessitated by the fishing constraints. The reality is that the operatives usually delegate duties to their subordinates when responsibilities and workload increase, but eventually, all processes have to be examined by them. In other words, they are overly burdened by additional workload for the fine detail of regulatory compliance but do not proactively invest in more advanced communication technologies. An example is given by Interviewee 9 who is responsible for preparing every port-in/port-out document and daily reporting to PIPO Control Center, and shares out the paperwork when the office gets busy, but eventually resumes responsibility:

*“I also delegate my responsibilities to my colleagues and teach them how to do my tasks. However, all processes and documents have to be ultimately verified and approved by me”* (Interviewee 9)

Entrepreneurs must maintain the environment surrounding them to be as stable as possible because defenders can perform well in the certain environments, and they are considered low in risk aversion.

As the EU IUU fishing regulations necessitate more than commitment, such as significant investment, in innovation adoption to support the sustainability objectives, the unfortunate choice of private fishers to choose low-cost fishing practices represents a position of *competition* (i.e. against legislative aims) within the Bavinck and Gupta (2014) categorization. Nonetheless, their “competitive” nature does support a slow but effective actual engagement in sustainability issues.

### Analyzers

Analyzers combine prospectors and defenders, thereby seek both stability and flexibility, and are adaptable to the uncertain environment, while maintaining some efficiency and cost-effectiveness. In this sense, the management of sustainability issues would on balance show signs of effectiveness. Private businesses, such as vessel owners and fish market entrepreneurs (e.g. Interviewees 5 and 7), seem to fall within this archetype because they have high potential to adapt their business to the new regulations, while remaining concerned about the costs. By this, we refer to their highly adaptive entrepreneurial skills and behavior. Different from the fishing and skipper companies of the defender archetype, vessel owners and the prominent fishing companies are more established and hold higher responsibility and bargaining power within the supply chain, and therefore are financially resourceful to support innovation. This level of support can satisfy the meaning of accommodation within the Bavinck and Gupta (2014) categorization. Since the new IUU fishing regulations were implemented, many fisheries have closed down, and vessels stopped operating due to their inadaptability of the new regulations and unaffordability of higher expenses. However, the stronger industry players survived, as an entrepreneur commented:

*“I have to adapt my business to follow the latest updated regulations suddenly by coordinating with other entrepreneurs … Also, due to an increase in operating costs, I have to keep other variable costs down… The most important thing is communication within the organization.”* (Interviewee 7)

Thus, the ability of the entrepreneur to carry out all the actions of additional external coordination, adapting to cost changes and additional internal communication is core to the analyzer archetype.

An analyzer’s performance is enhanced by scanning the environment before planning and taking actions and enabling prospectors to minimize uncertainty risks and maximize the opportunities for making a profit. For the fisheries context, when new regulations are enforced, the fishing owners should first attempt to understand the regulations intensively before responding with an action. Analyzers are more likely to imitate, but they systematically evaluate, new ideas before they move forward (see Jabnoun et al. 2004). Then, owners might learn from past experiences of a similar situation or from the industry/sector. This behavior seems to be the case for Interviewee 7, a fishing company owner.

### Reactors

Reactors perpetually reside in a stance of instability. Only the processing plant (Interviewee 6)—i.e. a stakeholder whose business is dependent on other effective stakeholders within the supply chain—showed closest attributes of a reactor. The plant indicated failure to respond urgently to changes in environmental conditions, such as the new fishing regulations, and the strategic responses available for the new working conditions:

*“Now, I’m forced to stop my business temporarily… My responsibilities do not change, but the only thing I can do right now is to take care of my assets and prepare to perform again”* (Interviewee 6)

The most crucial law that negatively affects the strategic performances of the plant processor is the three-nautical mile rule. The consequence of this is the volume of anchovy catch had decreased dramatically. This has directly led to revenue decline and incurrence of other expenses, but in turn has resulted in effective engagement with the required sustainability issue.

Staff involvement in decision-making can increase organizational effectiveness by enhancing mutual influence, motivation and satisfaction (Andrews et al. 2009). When employees feel that they are a substantial part of the organization, they will have genuine participation in any organizational activity. Occasionally, effective strategies are possibly contributed by operatives. Therefore, letting them participate in decision-making may be another possible way to avoid becoming reactors if the strategic view of firms is to avoid this archetype. Unfortunately, while processing plants (e.g. anchovies) are typically wealthy and operate a highly influential and important role within the industry in terms of price determination and financial crediting, they have suffered by the IUU fishing regulations and have little direct involvement in them. Thus, they are positioned at the lowest level of the fisheries hierarchy in terms of IUU fishing related environmental management. On the whole, given the ad hoc nature of reactors softens the impact of the effect of regulatory compliance on the business, it represents a position of indifference within the Bavinck and Gupta (2014) categorization, possibly because of the incontrollable position such a stakeholder is in. While business prospects are not encouraging for reactors, their unfortunate position has been the result of others engaging in effective (but not proactive) sustainability issues.

### (Mis)management of Sustainability Motivations

**Fig. 4:**
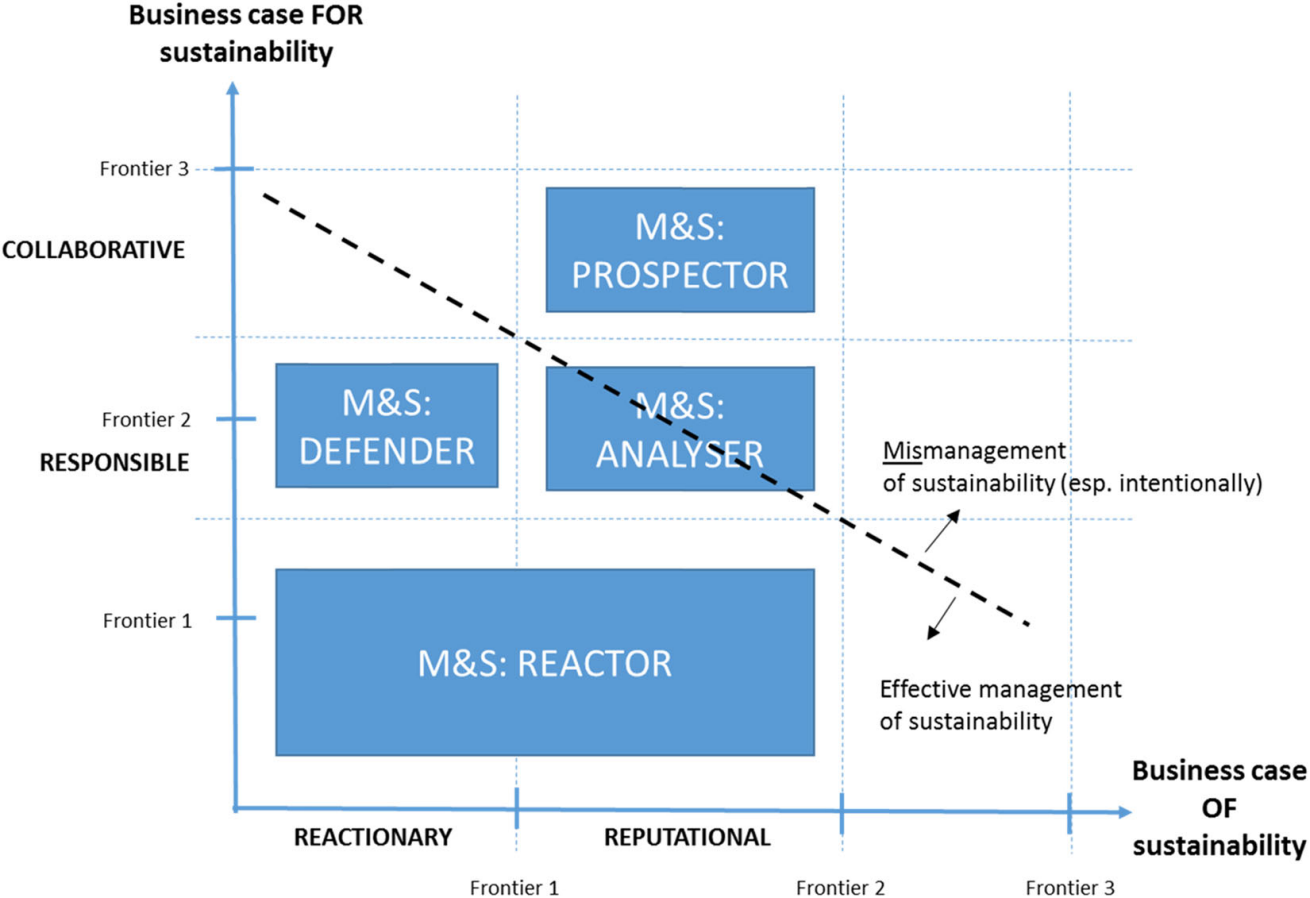
Miles and Snow archetypes within sustainability motivations and their management effectiveness

Following the research frames of effective/mismanagement of (Maniora 2018), and varying ethical motivations of engagement with (Schaltegger and Burritt 2018), sustainability, as introduced earlier in the present article, an integrated (composite) model of business archetypes (following Miles and Snow 1978) based on the Thai fishery sector, is hereby posited (see Fig. 4). The two axes of the figure present opposing situations of the “business case FOR sustainability” (*y*-axis, where engagement will likely bring profitability and economic success, because the involvement of firms is a responsible one, and often collaborative with authorities) and the “business case OF sustainability” (*x*-axis, where engagement is reactionary and reputational, for example to keep environmental critics at bay than for any obvious economic gain or financial benefit). Schaltegger and Buritt subtly suggested that firms can achieve positions bounded by these behaviors, thereby moving from one frontier to another. Hence these are used to form positions within Fig. 4 into which the four archetypes of Miles and Snow fit. We do so by drawing on the empirical evidence of our findings presented above. Note that the fitting of the stakeholder groups into the composite model is not based on their officially designated roles, but rather based on how they practically function from the empirical findings. Thus, seemingly “opposing” stakeholders (e.g. fisheries officers and vessel owners) may be positioned closely together within the composite model if in practice they achieve a similar sustainability objective. Recent research (e.g. Kadfak and Linke 2021) also recognized the importance of greater collaborative action and dialog among stakeholders, in spite of the power struggles.

Thus, relating to the “business case FOR sustainability” (*y*-axis), reactors are positioned within frontier 1, not reaching responsible or collaborative motivations, as identified from the empirical research. For example, an anchovy processing plant owner (Interviewee 6) remarked “I’m forced to stop my business temporarily”, suggesting a significant shortfall in seeing a business case for deliberately engaging in sustainability objectives. Defenders and analyzers fall within the “responsible engagement” frontier because, for example a defender “… delegate[s] … responsibilities to … colleagues and teach[es]”. In practice, some fishing companies can be a defender or reactor (depending on resources available to it and personal virtues about sustainability) so the financially stronger company may be positioned in frontier 2 (as a defender) in a position that sees a “business case” possibly for the longer-term survival of the business (e.g. better fish stocks in the future). A prospector however would collaborate with various authorities (falling within the “collaborative” position), for example as Interviewee 2 indicated above, to engage in the business activity. In this sense, the direct “business case” need (i.e. their job responsibility) places them in the highest position of the axis. As analyzers are a combination of prospectors and defenders, they therefore fall within the position in-between them in frontier 2. Such businesses are likely to see some “business case” for sustainability involvement but are comprised of smaller-scale fishers who lack the financial resources and drive to do so actively.

Relating to the “business case OF sustainability” (*x*-axis), defenders and reactors are “reactionary” to sustainability requests of the general environment; a typical captain on board (Interviewee 11) above indicated “… it is not easy to adapt working practices to every single regulation frequently and entrepreneurs probably cannot afford the higher costs”, suggesting a lack of appetite to respond even to legislation. Reactors are also “reputational”, as are analyzers and prospectors, as they are local businesses that would suffer from poor publicity, and governmental officials whose work must be sacrosanct and in full abidance with law. This coincidental alignment of (unlikely) stakeholders who are driven by reputational gains suggests that greater effort to work collaboratively on sustainability objectives may be beneficial. Voluntary and advisory schemes (see FAO 2022) have already started—such as the Coastal Fisheries Initiative and other similar diagnostic tools—have advised the closer working together or governance and local legislation to support smaller-scale fishers. These have yet to become extensively operative in this region.

Maniora’s (2018) notion of effective management and mismanagement of sustainability issues is represented by the broken line in Fig. 4, dividing the Miles and Snow archetypes into either the bottom left positions representing effective management or top right positions representing mismanagement of sustainability issues. The positioning of these archetypes is based on the empirical evidence above, in alignment with Maniora’s (2018) findings. For example, she argues that “prospectors are more likely to engage in the intentional mismanagement of sustainability than defenders” (ibid: p.10); this is consistent with the interviewee comments above. Similarly, as analyzers are theoretically both (or either) prospectors and/or defenders, they are positioned in-between the line representing occasional engagement with both effective management and mismanagement of sustainability issues. These stakeholders are familiar with the industry and can negotiate for short-term gains, thereby moving in a constant flux between effectively and ineffectively achieving sustainability objectives. Reactors might be weak in their financial position because of their slowness to engage with business affairs, but they are risk averse and constrained by the imposing environmental restrictions, thereby having already engaged/complied with the sustainability objectives. Therefore, they naturally fall within the notion of “effective management of sustainability”, close to that required by legislation, and perhaps without knowing it!

## Conclusion

The research has used several configuration principles to explore and present rich commentaries about the fishery sector in Thailand—both factual constraints about the practicalities and opinions from key stakeholders about the EU IUU fishing regulations and local implementation laws. Thus this article contributes to knowledge by integrating orientations of business strategy in the form of archetypes for the fishery sector into two distinctive motivations for engaging in sustainability projects (i.e. the business case for and of sustainability), and indicates their likely effective management and mismanagement. Through this conceptualization, and several salient managerial implications and recommendations for regulatory policy makers, we argue the existence of a paradox, which we believe is applicable to conditions into which sustainability success is less stable and subject to complex constraints.

### Contributions

The research has identified that different fisheries stakeholders are impacted by the IUU fishing regulations and local implementation laws in various ways—such as anchovy processors whose workload is restricted by catch within limited distances, skippers who have fishing license limits their use to a single gear, etc. Their work frustrations and financial impact on their livelihoods—such as fisheries that have shut down or increase in administrative paperwork for businesses still in operation—were of little consideration in either EU or local laws, in favor of environmental conservation and fish stock sustainability. The study has also matched the different impacts on the respective stakeholders to different configurational archetypes that trigger different strategic responses, and in this way posited a composite model to conceptualize the sustainability outcomes.

The composite model (Fig. 4) offers the key proposition: “as either the business case OF or FOR sustainability increases (i.e. diagrammatically moving across frontier 1, through frontier 2, to frontier 3 on either axes), we move toward organizational configurations resembling more closely to that of the prospector archetype, but the stakeholders’ management effectiveness of the sustainability issues decreases, moving from a categorization of “effective management” to one of “mismanagement” possibly with intention”. In other words, “when it makes more business sense for a fishery stakeholder to engage in sustainability matters, a higher quantity of engagement takes place, but of the wrong kind resulting in the true sustainability objectives poorly performed. Thus a paradox has emerged”. Specifically, fishery stakeholders that are more characteristic of prospectors (namely, legal officers)—positioned top-right of Fig. 4—pose greater risk of mismanaging the sustainability objectives in question. Stakeholders to the bottom left of the figure (e.g. defenders and reactors—such as vessel owners and fish processors) are not necessarily exempt from mismanaging sustainability, but the consequences of their inactive engagement—mainly due to lower financial resources—are unlikely to lead to significant dangers for which environmental sustainability objectives were designed to avoid.

### Managerial Implications and Recommendations

Fishing is a complex business and overall environmental sustainability is not easily within a business’s, or regulator’s, control. Practically, the IUU fishing regulations impact on different parts of the Thai fishery sector differently, and the stakeholders’ responses are dependent on the archetype into which they may be categorized. In the same vein, our composite model of configurational positions articulates a message that some archetypes are more effective than others in (truly) managing sustainability issues. The managerial implications for stakeholders concerned and recommendations for regulators/legislators, for softening the blow of impact, are as follows.

#### Regulators vs. legislators

So far, we have discussed the need to ensure more effective regulation/legislation as if regulation and legislation fall naturally together or that the terms can be used synonymously or interchangeably. However, our research has clearly recognized the role of regulation has belonged to the European Union in terms of the fisheries policies and that the Thai context of application impacted by the regulation concerns local laws and community cultures – i.e. that of legislation to implement the specific requirements. This means, the gulf between the two requires recognition of the role of the governance systems (such as role of government and legal officers) and the rule of law, with specific respect and consideration of local people. Given regulators are not legislators, the managerial implications for each are different. For the former, the role of regulation must be broad and embracing of global sustainability concerns, and regulatory standards can be broad-brushed to be comprehensive of all international waters for the common good, but their penalties need be more considerate of the local conditions they affect. In other words, sanctions and penalties need to be a case-basis. While it would be desirable to have individualized IUU fishing standards for the different areas of catch and processing, we do also need be wary this might arouse political tension among the international community. However, we believe stronger recognition of cultural differences and practical constraints can at least be acknowledged. For the latter, local legislators act as a conduit for the success of the regulatory standards and local laws must recognize the conditions of business operation among the stakeholders. To that end, it is necessary to adopt mechanisms that consult the industry players to understand how the laws take into consideration the finer detail of daily work. A common opinion of the interviewees has been the lack of connection between lawmakers and the businesses concerned, by both the content and process of local legislations. It is also imperative to clarify: the EU is not “regulation” per se (as it may have served that proxy for research purposes), but yet another stakeholder within the long chain of control. While “regulating” may be a principal function of the EU within its broader remit, it is therefore not infallible to making mistakes, and valuable lessons can certainly be learned by all stakeholders.

#### Hearing smaller stakeholders

Our research has opened up a broader group of stakeholders in the Thai fishery sector and has heard the voices of smaller players with unique impacts on their business and environmental behaviors, which to now has been under-presented in the literature (see Zeller and Pauly 2019). While smaller-scale fishers do have committees of representation, in practice they often lack the confidence to air their views (Kadfak and Antonova 2021) or see that they are too low in the hierarchy to be deemed important by the bigger stakeholders who represent them in price bargaining or take their other concerns to heart (FAO 2015). In the reluctance of all voices heard, information remains partial, and any attempt to implement policies or objectives can at best only target the voices that shout loudest, which in the fishery sector are those who are positioned higher up in the hierarchy and do not represent the operational activities. Without a significant shakeup to formal industry and sectoral mechanisms of communication, local communities can try to facilitate one another in the form of goodwill and collegiality and “spread the word”, so to speak, of any difficulties they may be facing because of changes in fishing standards. Through this, over time, cultural changes will likely lead to more formal mechanisms of community feedback to impact on future legislative control.

#### Closing the disconnect

We started our article by articulating the Thai fishing context as a black box, which forms the knowledge gap the research would fill. The existence of the gap represents a disconnect between regulators/legislators and frontline operators. Our empirical findings have outlined a number of nuances that were previously little known, and by addressing these can close that disconnect. For example, understanding of IUU fishing was varied between the stakeholders. Given the technicality of the definitions, some smaller fishers were illiterate and could not comprehend the technical details, while the larger firms were well aware of the details and had resources to prevent serious impact. In either case, the fishery stakeholders were impacted directly by the regulatory (or local legislative) constraint and less so by the overarching purpose of sustainability. The latter is likely to have a stronger natural alignment of effort at all levels than the finer details of associated objectives/standards. Hence, we believe a closer connection between the measures used—such as labor control, for example—to the objectives of IUU fishing. This way, even if the message of sustainability cannot be well communicated, the stakeholders may still see the benefit and put in a greater effort to comply with new requirements.

#### Rethinking the role of “effective” stakeholders

Our grouping of stakeholders into strategic archetypes was the outcome of understanding their general behavior. For example, legal officers/authorities were naturally prospectors due to duty and cost affordability, while others at the frontline of sustainability (e.g. fishers) were less proactive. Our extant knowledge determined a desire for stakeholders to be prospectors if they would benefit regulatory impact. If we insist this to hold true, we must concur with the need to make better/different use of them as enablers for achieving objectives. In the case of the Thai officials, this would require broadening legal officers’ role within society and business community, although in practice this may be seen as micromanagement and a conflict of interest in western eyes. If we are alternatively not dogged by the view that only prospectors, and their associated proactive behaviors, can result in strategic success, then we can turn our attention to stakeholders, say within the reactors archetype, who in our research are already engaging in the true sustainability issues. Such approach could involve direct financial assistance from the Thai government—such as compensation for losses caused by strict IUU fishing legislation, like subsidies and incentives programs, even for the short-term—until a clearer picture of progress emerges.

#### Defining sustainability

We have premised our research on a generic understanding of sustainability to expect good to fishers, global fish stocks, and society. However, sustainability has shown to mean differently for each of these groups. Good to fishers was understood to mean the ability to support a livelihood; good to global fish stocks means the effective management of fishing practices to restrict damage; and good to society means the harmonious co-existence of nature and all fishery stakeholders. This difference is because of the conflict of self-vested interest. It has not been possible to accomplish good to all these groups, and striking the fine balance has rested on the understanding of what sustainability means (and the importance of it attached) to each group. We argue the beneficial assembly of representation from these groups to offer up a more unified and agreed understanding of sustainability. By doing so, we may discard such labels as “true sustainability” or “sustainability issues”, and avoid any paradox altogether.

### Final Remarks, Limitations and Future Research

The present research has potential extendibility to understand conditions beyond Thailand and fishing—such as the post-Brexit future of the UK which is no longer governed by the EU Common Fisheries Policy which is currently evidencing a new fishing row, and must find a way to operate its own set of local legislation by understanding constraints and disconnections between the stakeholders while achieving sustainability targets (see Kemp et al. 2020). We acknowledge some methodological limitations of the study—such as the snowball sampling approach used for the breadth of interviewees selected, their representativeness and suitability which we assumed without extensive vetting, and location of research restricted to a specific (but very important) community of the Andaman coast. Further, our composite model of configurations was deduced against this backdrop, for mostly smaller stakeholders and associated authorities in a specific areas of Thailand’s fishing region. While this might restrict the generalizability of the model’s extensive application, this poses sound opportunities for future research to test empirically our assertions in other contexts of sustainability management.

We also recognize our stipulation of stakeholder impact, and subsequent grouping into strategic archetypes, were based on research with individuals who commented about the EU IUU fishing regulations’ impact on their work and generally about the “organization” (business, institution or legal authority) to which they belonged. We were therefore careful not to assume the direct employer-employee relationship in deriving our findings and employed the template data analysis technique that grouped commentary in context, and not respondents. Nonetheless we do caution our readers not to over-interpret the term “stakeholder” in categorizing strictly every person into groupings but to use our composite model as general guidance than a manual of instruction.

While this study has presented its findings and explained its methodology as objectively as possible, it is not without its limitations. For instance, the study did not explore how legislation may be implemented more effectively in order to benefit the reduction of conflicts and the way stakeholders receive it. Some of the interviewees believed fishing legislation and regulations in Thailand are effective sustainability measures, but the method of implementation is too forceful and aggressive. One possible reason for this could be that Thailand was too hasty in implementing new IUU fishing controls compared to other countries, so a different direction of exploring countries with slower law-making mechanisms may be beneficial. Possible research questions related this may be, for example: what kind of country benefits from the different types of legislative control?; what is the impact of different national cultures on fishery stakeholders?; and, are there more appropriate configurational approaches more suited for understanding environmental sustainability, as the world’s sustainability agendas become more aligned with fishery interest, such as global food supply security? Until we have answers to these future research questions, we hope the findings and theoretical contribution (i.e., compositive model) from the present study will elucidate why there exists a paradox of regulating environmental sustainability—that imposing more blanket legislation does not result smoothly in an increased level of sustainability.

## Appendix A: Interviewee Profiles

**Table:**

Interviewee	Position	Stakeholder	Years experience	Level	Responsibilities
1	Fisheries Officer	The Department of Fisheries, Satun Region	23	Middle	Operating officer at Port in–Port Out Control Center, port out inspector, control fishery documents
2	Marine Officer	The Marine Department, Satun Region	30	Middle	Operating officer at Port in–Port Out Control Center, enroll fishing vessels
3	Fisheries Officer	The Department of Fisheries, Satun Region	6	Middle	Operating officer at Port in–Port Out Control Center, port out inspector, control fishery documents
4	Legal officer (Practitioner level)	The Department of Labor Protection and Welfare	5	Middle	Operating officer at Port in–Port Out Control Center, protect both Thai and Non-Thai labors and welfare
5	Vessel owner and Captain on board	Thai Vessel (pseudonym)	19	Low	Fishing controller, supervise all activities on board as well as crews
6	Owner	Thai Anchovy Processing Plant (pseudonym)	12	Low	Supervise the whole company
7	Owner	Thai Fishing Company (pseudonym)	3	Low	Supervise the whole company
8	HR Manager	Thai Fishing Company (pseudonym)	20	Low	Recruit migrant workers, prepare MCPD documents, prepare daily catch report
9	Clerk	Thai Fishing Company (pseudonym)	23	Low	Prepare and approve official documents regarding IUU Fishing Regulations
10	Captain on board	Thai Fishing Company (pseudonym)	30	Low	Skipper
11	Captain on board	Thai Fishing Company (pseudonym)	30	Low	Skipper
12	General Manager	Thai Fishing Company (pseudonym)	25	Low	Employee controller, prepare the customers’ order, price negotiator
13	Marine Police First Lieutenant	The Marine Police Division	25	Middle	Operating officer at Port in–Port Out Control Center, rescue the Thai territorial waters, port out inspector
